# Opioid Prescription Consumption Patterns After Total Joint Arthroplasty in Chronic Opioid Users Versus Opioid Naive Patients

**DOI:** 10.5435/JAAOSGlobal-D-20-00066

**Published:** 2020-06-16

**Authors:** Austin C. Kaidi, Akshay Lakra, Emma L. Jennings, Alexander L. Neuwirth, Jeffrey A. Geller, Roshan P. Shah, H. J. Cooper, Thomas R. Hickernell

**Affiliations:** From the Department of Orthopedic Surgery, Columbia University Medical Center, New York, NY.

## Abstract

Although chronic preoperative opioid use has been linked to inferior total joint arthroplasty outcomes, little research exists on postoperative prescribing patterns for opioid-naive orthopaedic patients versus chronic opioid users.

**Method:**

The New York State Prescription Monitoring Program database, which tracks controlled prescriptions dispensed in-state over the preceding 12 months, was retrospectively queried for 386 patients who underwent primary, elective total hip or knee arthroplasty at a high-volume, urban medical center from May through December 2017. Seventy-four patients were excluded because they did not return prescription monitoring program results, leaving 312 patients. Prescribers, medications, dates prescribed and filled, and quantity dispensed were recorded 3 months preoperatively through 12 months postoperatively. We defined chronic users as ≥2 opioid prescriptions filled in 3 preoperative months and opioid-naive as <2 filled. Opioid use was compared univariately using 2-tailed Student *t*-tests.

**Results:**

Chronic opioid users (n = 49; 15.7%) filled an average of 13,006.64 morphine equivalent doses per patient in the 12-month postoperative period, while opioid-naive users (n = 263; 84.3%) filled an average of 854.48 morphine equivalent doses per patient (*P* < 0.01). Opioid use in the chronic-user group was significantly higher in each 6-week postoperative interval (*P* < 0.01). These trends remained significant when stratified by procedure. For opioid-naive patients, 74% of opioid prescriptions were prescribed by our orthopaedic department. For chronic users, only 21% of opioid prescriptions originated from our department. Chronic users were found to cyclically fill opioid prescriptions every 3 to 4 weeks postoperatively as far out as 12 months and were significantly more likely to fill nonopioid controlled substance prescriptions both preoperatively and postoperatively (*P* < 0.01).

**Discussion:**

Chronic opioid users undergoing arthroplasty filled significantly more opioid prescriptions than opioid-naive patients. Chronic users obtained prescriptions from myriad sources, only a minority of which originated from our orthopaedic department. In the current opioid epidemic, vigilance regarding opioid prescribing is critical.

Prescription opioid misuse in the United States has reached epidemic levels.^1^ Among prescribers, orthopaedic surgeons rank in the top five specialties prescribing opioids.^2^ Total hip arthroplasty (THA) and total knee arthroplasty (TKA) surgeries are among the most commonly performed orthopaedic procedures and typically require opioid pain management postoperatively. Currently, over one million total joint arthroplasties (TJAs) are performed annually in the United States.^3^ These rates are projected to rise over the next several decades. Prescription opioid medications are part of most standard postoperative pain control regimens after TJA but are also sometimes prescribed in the preoperative period to manage osteoarthritis-associated pain. Overprescribing of these opioids can have multiple harmful medical, social, and legal ramifications for the patient and for the prescriber.

A large volume of research has examined the effects of chronic opioid use on outcomes of TJA,^4,5,6,7,8^ and many studies have proposed methods to optimize perioperative inpatient pain controls with multimodal analgesia to minimize the need for opioids.^9,10,11,12,13,14,15,16,17,18^ Relatively little work, however, has been done studying best practices for prescribing opioids to control acute pain in the TJA postdischarge period. Although studies and anecdotal evidence suggest that most patients require at least 3 to 6 tablets of oxycodone (5 mg) per day for the first 1 to 4 days after TJA,^16,19^ the optimal dose and the length of opioid prescription remain poorly defined.

To combat opioid overprescribing, most states now require electronic prescribing for all controlled substances. In addition, statewide prescription databases have been developed to allow physicians to more easily identify patients and prescribers with at-risk behaviors and to prevent “doctor shopping,” when patients find multiple physicians to prescribe them opioids for the same issue. These prescription monitoring programs (PMPs) have served as helpful tools for examining controlled substance use patterns. These mandatory databases have also been used by previous groups to gain further insight into opioid prescribing patterns in various groups of patients.^20-22^ These PMP databases only provide information on controlled substance prescriptions (ie, opioids, benzodiazepines, hypnotics, and cannabinoids). Physicians are often only able to search these databases in the state which they are certified to practice medicine.

The purpose of this study was to use the NY State prescription monitoring system to evaluate the rates of preoperative and postoperative opioid prescription use in patients undergoing TJA at one high-volume, urban academic medical center. The goal was to better understand current patient opioid use and our patients' sources of preoperative and postoperative opioid prescriptions.

## Methods

### Study Design

Approval was obtained from our institutional review board before commencement of any study-related activities. A retrospective chart review was performed for all patients who underwent primary, elective TKA or THA at our urban academic medical center from May 1, 2017, to December 12, 2017. Patients undergoing unicompartmental knee arthroplasty, revision arthroplasty surgery, or bilateral surgery were excluded.

Patient demographics including age and sex were recorded. Using unique patient identifiers, the New York State PMP was accessed to determine the quantity of controlled substance prescriptions filled by each patient in the 3-month preoperative and 12-month postoperative periods. This preoperative window was in line with previously published studies and allowed for characterization of preoperative opioid use patterns.^20,21,23^ The 12-month postoperative period allowed for a full characterization of opioid use and long-term trends. As the New York State PMP only reports the most recent 12 months of data within the search date, the database was queried longitudinally over a 12-month period to gather ongoing postoperative prescription patterns for this same cohort of patients. The PMP was last queried in December 2018. Of note, patients without entries in the PMP were also excluded because they were assumed to reside or fill prescriptions outside the state of New York.

The prescriber, medication, date prescribed, date filled, and quantity prescribed were recorded from the PMP. When unknown, prescriber information was identified by internet search.

### Statistical Procedures

Patients were stratified by THA versus TKA patients and opiate naive versus chronic users. Chronic users were defined as patients who had filled two or more opioid prescriptions in the 3-month preoperative period, which is consistent with previous studies.^20^ This number was chosen to prevent overclassification of patients as “chronic” opioid users when it is not uncommon to get low-dose, one-time opioid prescriptions for dental procedures, acute trauma, etc. All opioid prescriptions were quantified by morphine equivalent doses (MEDs), also known as morphine milligram equivalents, using the NYC Health Morphine Milligram Equivalent Calculator,^24^ which utilizes literature standards for opioid conversion.^25^

For quantitative analysis, opioid use was stratified into 6-week windows for the preoperative and 6-month (24-week) postoperative periods so that usage could be trended. One-year postoperative data were analyzed using 3-month windows for ease of data presentation. Data were ensured to be parametric before continuing analysis. Each group's opioid use was then compared univariately using a 2-tailed Student *t*-test. Heteroscedasticity was assumed for each sample. *P* values of 0.05 or less were considered statistically significant. Opioid use was also qualitatively assessed for each week pre-TJA and post-TJA and shown as average MEDs filled per patient.

## Results

### Demographics

Three hundred eighty-six patients who underwent primary THA or TKAs were identified by using the chart review. Seventy-four patients were excluded because they did not return results in the PMP and were assumed to reside out of state or fill prescriptions out of state. Of the remaining 312 patients, 179 (57.4%) underwent TKA and 133 (42.6%) underwent THA. Demographic data were reflective of a typical arthroplasty population (Table 1).

**Table 1:** Demographics

Demographic Group	TKA (n = 179)	THA (n = 133)	All (n = 312)
Age (mean ± SD)	69.5 (±8.52)	64.0 (±12.39)	67.2 (±10.68)
Female (N)	119 (67%)	69 (52%)	188 (60%)
Male (N)	60 (34%)	64 (48%)	124 (40%)
Opiate naive (N)	157 (88%)	106 (80%)	263 (84%)
Chronic opiate users (N)	22 (12%)	27 (20%)	49 (16%)
With pre-op nonopioid prescription	23 (13%)	22 (17%)	45 (14%)
Without pre-op nonopioid prescription	156 (87%)	111 (83%)	267 (86%)

THA = total hip arthroplasty, TKA = total knee arthroplasty

### Perioperative Opioid Use

A total of 1,881 prescriptions for controlled substances were captured in the NYS PMP database during the study period, which included the 3-month preoperative and 12-month postoperative windows for each patient. One thousand two hundred thirty-six (65.7%) of these were opioid prescriptions, most commonly (1) oxycodone HCl 5 mg (13.8%), (2) oxycodone-acetaminophen 5 to 325 mg (13.8%), (3) hydrocodone-acetaminophen 5 to 325 mg (8.6%), (4) tramadol HCl 50 mg (6.0%), and (5) acetaminophen-codeine #3 (1.4%) (Table 2). When converting opioid prescriptions to MEDs, the average prescription was written for 869.81 MEDs, equivalent to approximately 116 5 mg oxycodone tablets. The average prescription written by orthopaedic surgeons and orthopaedic staff was 423.71 MEDs, equivalent to approximately 56 5 mg oxycodone tablets. This is significantly higher than standard protocolled prescriptions. The average was brought up by 1-month pain management prescriptions prescribed by our teams.

**Table 2:** Most Common Opioid Prescriptions (n = 1,236)

Medication	No. of Prescriptions	MED/Prescription Mean	MED/Prescription SD	Intended Days/Presc. Mean	Intended Days/Presc. SD
Oxycodone HCl 5 mg	260 (21%)	564.66	308.24	11.72	7.21
Oxycodone-acetaminophen 5-325 mg tablet	259 (21%)	414.24	164.59	8.70	5.34
Hydrocodone-acetaminophen 5-325 mg tablet	162 (13%)	252.44	98.34	9.12	6.39
Tramadol HCl 50 mg tablet	113 (9%)	259.56	151.08	13.74	9.25
Acetaminophen-codeine #3 tablet	26 (2%)	193.20	100.30	9.88	7.06

MED = morphine equivalent dose

**Figure 1:**
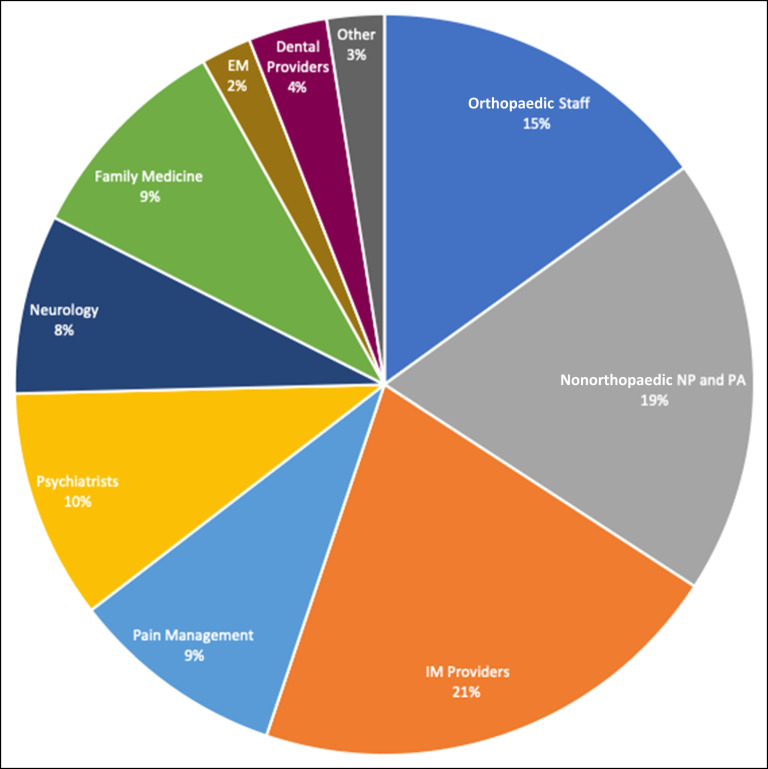
Chart showing preoperative opioid prescriber specialties for all patients. NP = Nurse Practitioner, PA = Physician's Assistant, IM = Internal Medicine, EM = Emergency Medicine.

**Figure 2:**
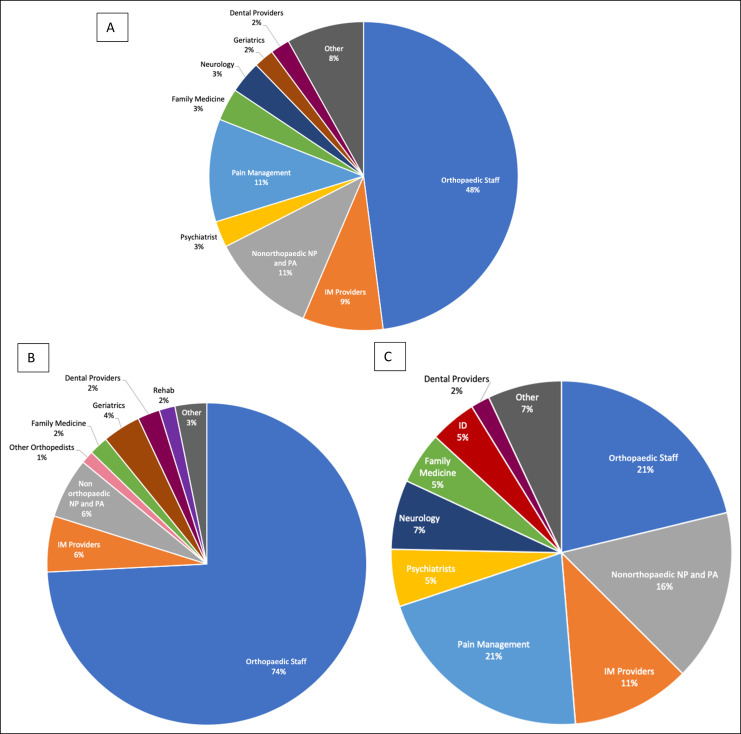
Chart showing (**A**) 12-month postoperative opioid prescriber specialties for all patients, (**B**) 12-month postoperative opioid prescriber specialties for opioid naïve patients, and (**C**) 12-month postoperative opioid prescriber specialties for chronic patients. NP = Nurse Practitioner, PA = Physician's Assistant, IM = Internal Medicine, EM = Emergency Medicine.

When looking at preoperative prescriptions, only 15% came to our staff. Nine percent came from pain management specialists, and the rest were from a variety of medical professionals (Figure 1). Overall, only 48% of all postoperative opioid prescriptions were written by our orthopedic staff (attendings, residents, nurse practitioners [NPs], physician's assistants [PAs]) (Figure 2, A); however, this differed with previous opioid use. Among the opioid naive group, our orthopaedic staff wrote 74% of the filled postoperative opioid prescriptions (Figure 2, B). Among the chronic group, our orthopaedic staff wrote only 21% of filled postoperative opioid prescriptions (Figure 2, C).

**Figure 3:**
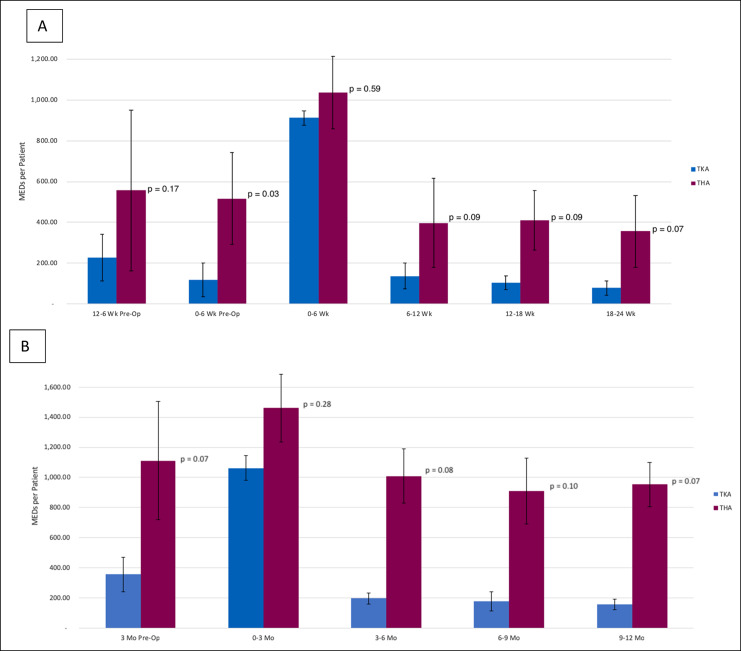
Chart showing perioperative opioid use for total knee arthroplasty versus total hip arthroplasty patients in the (**A**) 6-month postoperative time frame and (**B**) 12-month postoperative time frame. MED = morphine equivalent dose, THA = total hip arthroplasty, TKA = total knee arthroplasty

Among THA and TKA patients, in the 6-week preoperative period, THA patients on average filled 517.14 MEDs, while TKA patients filled 119.17 MEDs (*P* = 0.03). THA patients also tended to have higher opioid use in the entire 12-month postoperative period although this was not significant throughout (Figure 3).

**Figure 4:**
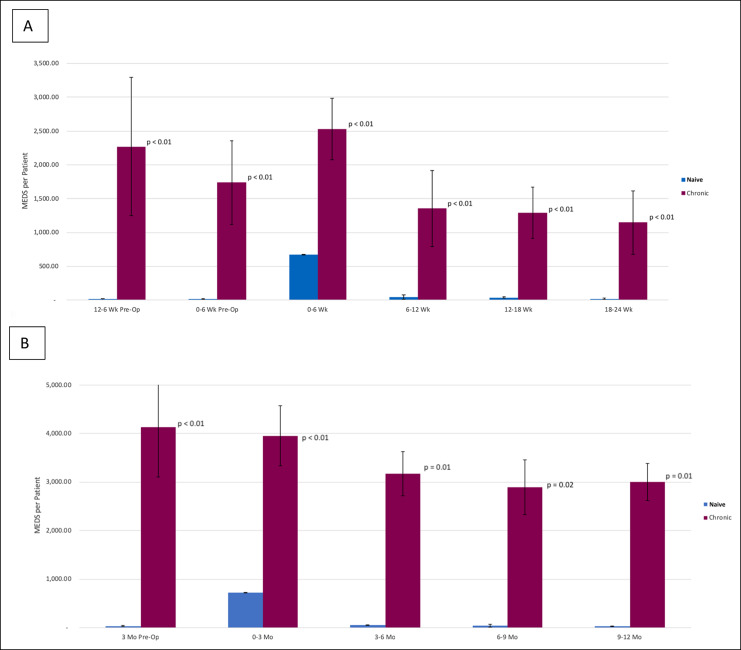
Chart showing perioperative opioid use for naive versus chronic patients in the (**A**) 6-month postoperative time frame and (**B**) 12-month postoperative time frame. MED = morphine equivalent dose

**Figure 5:**
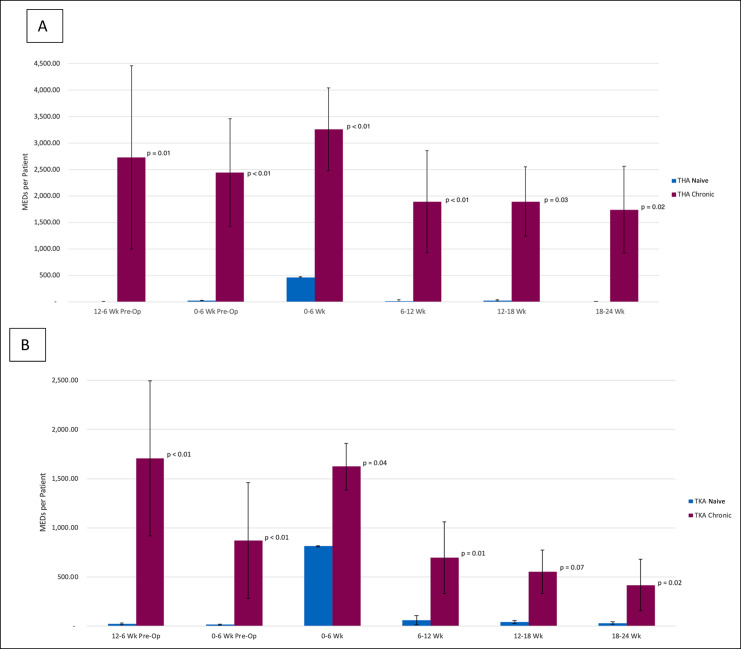
Chart showing perioperative opioid use for naive versus chronic patients in the 6-month postoperative time frame for (**A**) total hip arthroplasty patients only and (**B**) total knee arthroplasty patients only. MED = morphine equivalent dose, THA = total hip arthroplasty, TKA = total knee arthroplasty

Among chronic users and opioid naive patients, patients using chronic opioid exhibited higher opioid use in the entire 12-month postoperative period. Chronic opioid users filled an average of 13,006.64 MEDs per patient in the 12-month postoperative period, while opioid naive users filled an average of 854.48 MEDs per patient (*P* < 0.01). In the initial 6-week postoperative period, chronic opioid users filled 2,526.61 MEDs per patient compared with opioid naive patients, who filled 673.24 MEDs (*P* < 0.01). In the 6- to 12-week postoperative period, chronic users filled 1,355.97 MEDs, while opioid naive patients filled only 41.85 MEDs per patient on average (*P* < 0.01). In the 12- to 18-week postoperative period, chronic opioid users filled 1,257.38 MEDs more per patient on average (*P* = 0.01). In the 18- to 24-week postoperative period, chronic opioid users filled 1,129.80 MEDs more per patient on average (*P* < 0.01) (Figure 4). These significant differences in chronic and opioid naive users were maintained when stratified into patients who underwent TKA and THA (Figure 5). Postoperative opioid use was higher among chronic users as far as 12 months out (*P* < 0.01) (Figure 4). No statistically significant differences were observed in opioid use between the preoperative and postoperative periods for chronic opioid users.

Chronic opioid users were also more likely to fill preoperative prescriptions for nonopioid controlled substances, such as benzodiazepines and hypnotics. Twelve percent of opioid naive users had a preoperative, nonopioid controlled substance prescription, whereas 29% of chronic users did (*P* < 0.01). In the postoperative period, 15% of opioid naive users had a preoperative, nonopioid controlled substance prescription, whereas 25% of chronic users did (*P* < 0.01).

**Figure 6:**
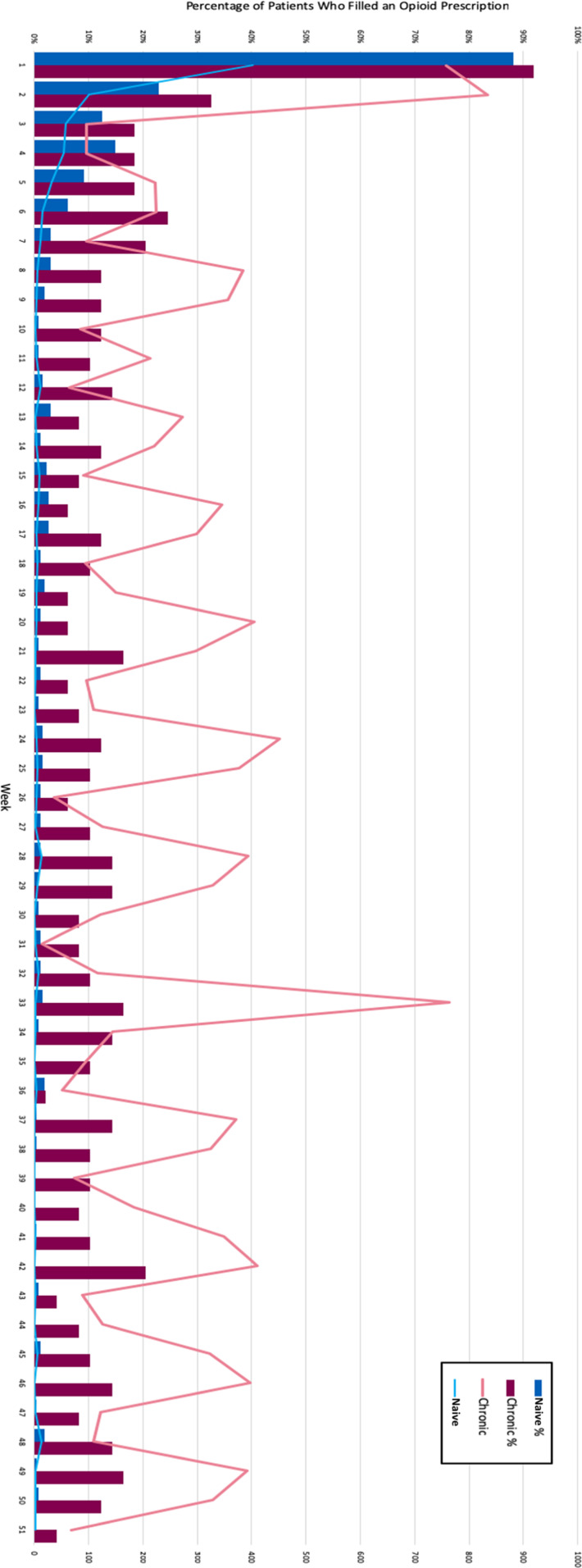
Chart showing naive versus chronic patient postoperative opioid use patterns by week. Bars represent percentage of patients who filled a postoperative opioid prescription per week. Line represents average morphine equivalent doses filled per patient per week.

More than 85% of both chronic and opioid naive patients filled an opioid prescription in a NY pharmacy in the first postoperative week. The percentage of patients filling prescriptions in each week thereafter dropped significantly. A greater portion of chronic opioid users filled an opioid prescription in each postoperative week (Figure 6). The average MEDs filled per patient in each post-op week shows chronic users filling consistently more prescriptions throughout the study period. Chronic users' prescription filling patterns oscillated, with peaks occurring ever 3 to 4 weeks postoperatively.

## Discussion

Opioid misuse in the United States has reached epidemic proportions, and orthopaedic surgery is one of the specialties leading to opioid prescriptions. For this reason, it is critical to understand the perioperative opioid prescribing and usage patterns for different patients and procedures. Better understanding of our TJA patients' opioid needs and patterns of use will allow for more judicious, safe, and effective pain management protocols to be developed and widely implemented. Many states, including New York, have implemented online digital PMPs to better track prescriptions and disbursements of controlled substances. Their aim is to give providers a better understanding of patients' potentially injurious patterns of use that may result from patients receiving opioid prescriptions from multiple providers. Currently, limited research exists in this topic.

One study^20^ performed in Michigan examined 315 consecutive primary TJA patients over a 13-month period, reviewing charts with the Michigan state PMP to document preoperative and postoperative prescription opioid use. The study found that at 1 year post-op, 64% of the chronic preoperative opioid users were still being prescribed opiates, compared with 22% in the control, or nonchronic user, group. Furthermore, 77% of all opiate prescriptions in the study were not prescribed by any practitioner within the orthopaedic group. This study also found no significant difference in perioperative use between THA and TKA patients. A similar study using Kaiser Permanent's Total Joint Replacement Registry and other Electronic Medical Record (EMR) found that in 48,000+ TJA patients done across the American Southwest, primary care providers were the highest prescribers of opioids in the preoperative and postoperative periods.^26^

Another study^21^ examined a large Humana database of 70,000+ patients who underwent TKA, assessing monthly opioid prescription filling rates in the 3-month preoperative and 12-month postoperative periods. They found that about one-third of patients used opioids preoperatively, with that rate increasing over the 2007 to 2014 study period, and that preoperative opioid use was predictive of having increased refills of opioids after TKA. A similar study of the Humana database obtained similar results.^23^

Yet another group sought to answer whether patients who received greater quantities of opioids after TKA were more likely to request refills.^22^ They found that rates of refills did not significantly differ between patients receiving smaller prescriptions and larger opioid prescriptions (>1,400 mg morphine equivalents per day [MED]).

Within our data, prescribing behavior was relatively consistent both in the selection of opioid and in the intended length of prescription. Sixty-six percent of all opioid prescriptions were for only the top five opioids and doses (Table 2). All of these were intended for an average of for 1 to 2 weeks of use. Although MED values may seem high to today's prescribers, this reflects the rapid change in prescription patterns that have occurred over the past few years. The MEDs per prescription also varied significantly. These differences could not entirely be explained by the difference between knee and hip arthroplasty. Except for the first 6 weeks postoperatively, no statistically significant differences were found in opioid use postoperatively between THA and TKA patients. Preoperatively, the THA cohort also had more chronic opioid users (20% versus 12%) and filled significantly more opioid prescriptions in the 0- to 6-week pre-op periods (*P* = 0.03). We also found that chronic users were, overall, more likely to fill more opioid prescriptions as far as 1 year after TJA, consistent with the existing literature.^18^ This may reflect concurrent hip and spine pathology. Although not statistically significant, postoperative opioid requirements for chronic opioid users trended down. This indicates that TJAs may be an important tool when fighting opioid dependence in those with chronic pain.

This study may contribute valuable information to the opioid literature by fully characterizing the physician specialties providing opioids not only in the postoperative period but also preoperatively. In both the preoperative and postoperative periods, orthopaedic staff are prescribing less than half of opioid prescriptions filled by TJA patients. In the preoperative period, they prescribed only 15%, and in the postoperative period, they prescribed only 48%. This difference was exaggerated when looking at the chronic opioid users. This indicates the need for an interdisciplinary approach to reducing opioid use for dealing with the chronic pain of hip and knee osteoarthritis.

In addition, this study qualitatively described weekly opioid use habits for our patient population, giving providers a more granular understanding of use patterns. We found that chronic users had cyclical opioid use patterns postoperatively and that this cyclical pattern was “reset” after the TJA with spikes in opioid refills every 4 to 5 weeks postoperatively. This indicates that orthopaedic surgeons have some influence on long-term patient opioid use patterns.

This study's utilization of a state-run database allows for recording of prescriptions that may have been missed by existing studies using only healthcare system databases and EMRs. For example, prescriptions from private pain management clinics, which made up a significant portion of prescriptions in our patient population, would have been missed using our health system's EMR. Furthermore, very few studies have described perioperative opioid use after TJAs in the Northeastern United States. This study demonstrated that TJA populations in this region have similar opioid use patterns described elsewhere in the United States in terms of chronic use and prescriber type.

We note several limitations to our study's methodology. PMPs are generally managed by individual states and do not return results for controlled substance prescriptions filled in nearby states. This proves problematic in border locations, where patients often seek care from physicians in neighboring states. In this study, 19% of patients did not return results in the NYS PMP, which could introduce follow-up bias. Recently, the National Association of Boards of Pharmacy's created PMP InterConnect,^27^ which would allow for multistate prescription look-ups. This could serve as a more efficacious way for surgeons to determine patient perioperative opioid use to minimize patient discomfort and excess opioid prescribing. In addition, the NYS PMP is limited only to a 12-month data collection window. Data must be aggregated over time to gain a complete understanding of patient use patterns. Additional limitations for this study include incomplete understanding of associated indications and outcomes. The NYS PMP does not include indications for prescriptions (eg, whether the prescription was written for postoperative pain after TKA versus written for chronic low back pain). Finally, clinical outcome data were not included for analysis although this is a target for further research.

Overall, our patients' continued use of opioids up to 1 year postoperatively indicates a significant need for better patient and provider education regarding the use of opioids for musculoskeletal pain management. A significant number of our patients who received TJAs were already chronic users—possibly secondary to the significant pain that advanced osteoarthritis can cause. Patients should be educated on other options for chronic pain management and when to seek an orthopaedic surgeon for more definitive treatment. If patients are already chronic opioid users, they should be preoperatively educated on expected postoperative opioid needs and options for opioid discontinuation if they encounter difficulties with persistent pain or addiction. Providers should be educated on the use of opioids for chronic management and on appropriate times to refer patients with osteoarthritis to an orthopaedic surgeon. Prescribers should be vigilant when continuing opioid prescriptions for musculoskeletal pain even after a patient has received TJA. Only with better patient and provider education will we be able to combat unnecessary use of opioids to treat musculoskeletal pain.
